# Acute pneumonitis and diffuse alveolar hemorrhage secondary to silicone embolism: A case report

**DOI:** 10.1097/MD.0000000000020578

**Published:** 2020-06-12

**Authors:** Alejandro Bejarano, Diego F. Bautista, Luz F. Sua, Bladimir Pérez, Juliana Lores, Marisol Aguirre, Liliana Fernández-Trujillo

**Affiliations:** a Department of Critical Care Medicine; b Faculty of Health Sciences, Universidad Icesi; c Department of Pathology and Laboratory Medicine; d Clinical Research Center; e Department of Internal Medicine, Pulmonology Service, Interventional Pulmonology, Fundación Valle del Lili, Cali, Colombia.

**Keywords:** alveolar hemorrhage, pulmonary embolism, respiratory failure, silicone

## Abstract

**Rationale:**

Polydimethylsiloxane, commonly referred as silicone, is an inert liquid compound used in esthetic procedures due to its durability and thermal stability, yet the application of non-pure silicone generates risks. One of the complications is systemic embolism syndrome which is presents with fever, hypoxemia, and progression to respiratory failure, diffuse alveolar damage and alveolar hemorrhage, as well as neurological alterations in one-third of the cases. Management is strictly supportive. We present the case of acute pneumonitis with alveolar hemorrhage after silicone injection.

**Patient concerns:**

25-year-old transsexual man, who consulted 48 hours after liquid silicone injection in the buttocks and trochanteric area, with progressive dyspnea and chest tightness, with rapid progression to respiratory failure.

**Diagnosis:**

Clinical diagnosis of silicone embolism was made. Chest x-ray and CT angiography showed diffuse alveolar infiltrates and pleural effusion without evidence of acute venous thromboembolism. Bronchoscopy plus bronchoalveolar lavage showed hemorrhagic fluid, 60% macrophages with hemosiderin in cytology and negative cultures.

**Intervention:**

Sedation, relaxation, pronation, and protective ventilation were implemented until hemodynamic stabilization; as well as IV steroids and antibiotics.

**Outcomes:**

Clinical progress was slow towards improvement with resolution of radiological or physical abnormalities. Despite severity, the patient improved satisfactorily without late sequelae.

**Lessons:**

Silicone injection can trigger phenomena similar to that seen in fat embolism causing inflammation and immune response activation that lead to alveolar hemorrhage, diffuse alveolar damage, and acute respiratory distress syndrome. We reported pulmonary complications related to the illegal use of injected silicone for esthetic procedures.

## 1 Introduction

Silicone is a chemically inert compound used for esthetic procedures due to its durability and thermal stability.^[1]^ In its pure form it has been used for >6 decades with minimal tissue reactions and without acute, immune, or granulomatous manifestations as long as the recommendations for its use are guaranteed and the application of any of its non-medical variations are avoided.^[1]^

Advances in plastic surgery and esthetic medicine have resulted in the frequent use of silicone in both developed and developing countries.^[2]^ According to the ISAPS (International Society of Aesthetic Plastic Surgery, report 2018) during 2017 >23 million esthetic procedures were performed worldwide, being Botox application for eliminating wrinkles and expression lines the most common, followed by silicone implants in the breasts, buttocks, trochanteric areas, and vaginal walls.^[3]^

Trends in plastic surgery vary according to geographical regions. In Latin America, in countries such as Colombia, Argentina, and Brazil, culture preferences tend to favor augmentation of the buttocks, breasts, and application of trochanteric silicone; while in Anglo-Saxon countries facial plastic surgery such as rhinoplasty predominates. Clandestine application of intramuscular and subcutaneous industrial silicone performed by non-certified personnel is increasing, generating risks for patients, especially in vulnerable populations such as transsexual individuals.^[4]^

In Colombia, the number of procedures is underestimated due to underreporting and lack of control in the sale of industrial silicone for illegal surgical procedures. The preferred sites for silicone injection are the breasts, buttocks, trochanteric area, and vaginal wall. The use of silicone is not exempt from complications especially if used impure. Adverse reactions due to systemic migration have been reported. Symptoms may include dyspnea, chest pain, fever, cough, hemoptysis, diffuse alveolar damage, pneumonitis, and acute respiratory distress syndrome. In addition, long-term liver alterations have been described, as well as lung and kidney disease due to autoimmune and granulomatous reactions, and severe local lesions. All of the above tend to increase when the silicone used is intended for industrial and not medical use.^[5,6]^

We report the case of a transsexual male patient who, following injection of liquid silicone, presented severe respiratory distress syndrome, pneumonitis, and severe diffuse alveolar hemorrhage.

## 2 Case report

**Figure 1:**
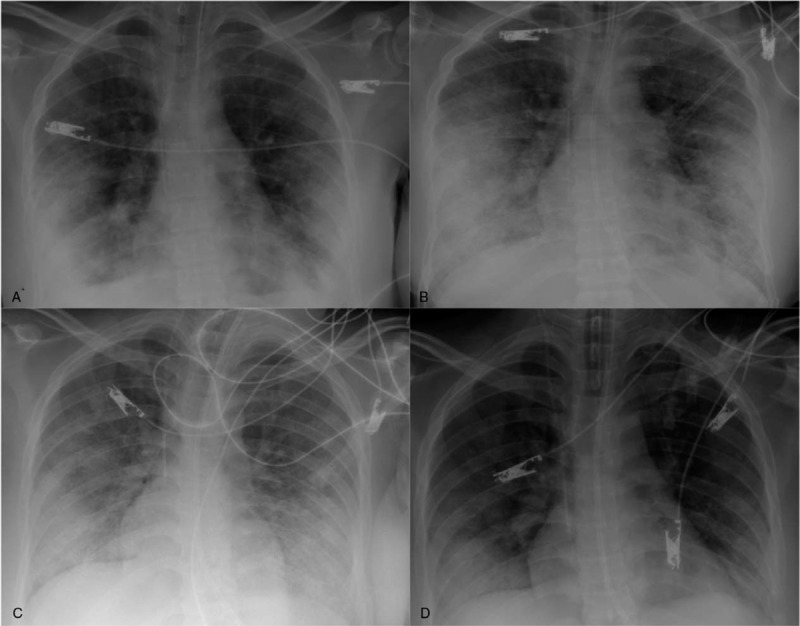
A–D. Chest x-ray evolutionary in time from projection A showing bilateral basal patchy ground glass infiltrates and pleural effusion. B and C. Worsening of the infiltrates occupying the lower two-thirds with greater profusion of the infiltrates of alveolar occupation. D. Chest x-ray with decreasing infiltrates remaining in the bases, which corresponds to the final days of mechanical ventilation.

**Figure 2:**
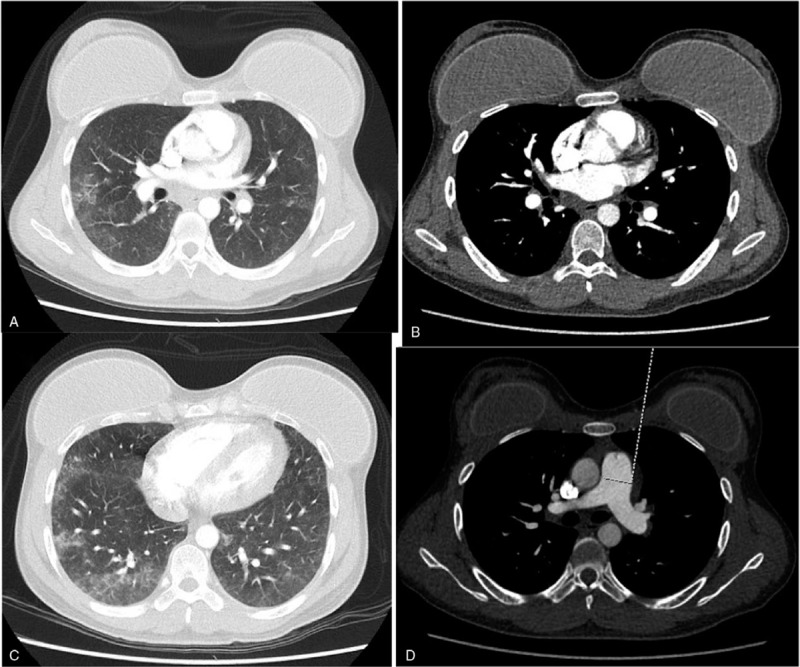
A and C. Thoracic CT angiography, lung window, with multiple infiltrates of alveolar occupation accompanied by ground glass patches, presence of breast prostheses. B and D. Mediastinal window showing pulmonary circulation without evidence of venous thromboembolism and aspect of the normal pulmonary artery. CT = computed tomography.

25-year-old transsexual male patient, with history of previous esthetic surgical procedures (rhinoplasty and mammoplasty), who consulted to the emergency department with 48 hours of progressive dyspnea, fever, cough, and chest tightness after undergoing industrial liquid silicone injection in the buttocks and both trochanteric areas. On admission the patients was in respiratory distress, with BP 111/58 mmHg, HR 121/bpm, RR 21/Bpm, SaO2 98% with FiO_2_ of 32%, no neck masses, no jugular engorgement, rhythmic tachycardic heart sounds without murmurs or gallop, decrease in respiratory sounds in both bases and normal abdominal examination. Areas of erythema, localized heat and swelling of approximately 10 cm were found in the buttocks and hips. Neurological examination was normal. Laboratory tests showed leukocytes: 12.370/mm, hemoglobin: 11 g/dL, platelets: 231,000/mcL. Arterial blood gases showed pH 7.4, pCO2 38.6, pO2 97, SaO2 97%, base excess (BE)—0.6, HCO3 23.5. He had normal serum sodium, potassium and chloride, and normal kidney function. Chest x-ray (Fig. 1A–D) showed bilateral patchy alveolar infiltrates mainly in the bases, and right-predominant bilateral pleural effusion. Thoracic CT angiography (Fig. 2A–D) showed multiple patchy infiltrates of alveolar occupation with extensive areas of ground glass opacities without alterations in the mediastinum, and no evidence of acute thromboembolism.

The patient presented a torpid evolution, with an increase in respiratory work during the next 8 hours, referring severe dyspnea. He was polypneic with a progressive decrease in oxygen saturation despite high supplemental oxygen. In addition, there was a 2-g decrease in hemoglobin. He underwent orotracheal intubation and was transferred to the ICU. Antibiotic therapy with clindamycin, cefepime, and vancomycin was initiated due to suspicion of soft tissue infection in the injection areas, and methylprednisolone was added. During the next 12 hours, mechanical ventilation became more difficult with increased peak pressures. Muscle relaxation was initiated in addition to previous sedation; positive pressure was titrated at the end of PEEP expiration based on lung mechanics avoiding plateau pressures >28 cm H_2_O. Prone position was implemented in 18-hour cycles, with progressive improvement of oxygenation and ventilation parameters.

**Figure 3:**
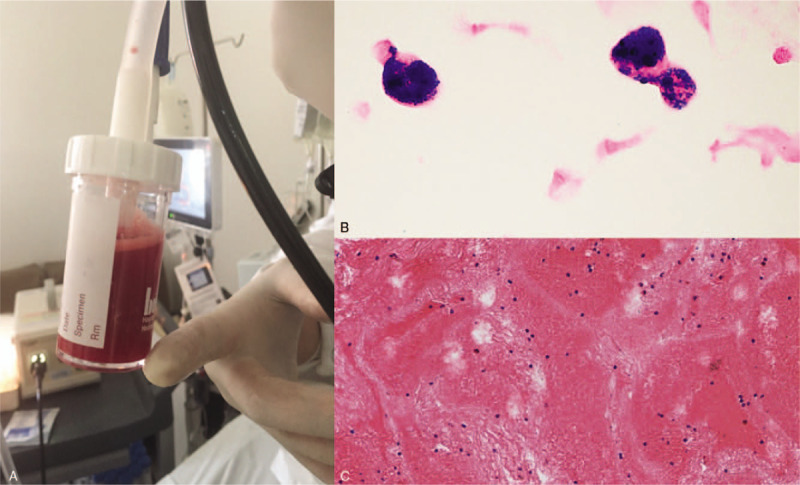
A. Grossly hemorrhagic macroscopic appearance of the fluid recovered during bronchoalveolar lavage. B. Cytology of bronchoalveolar lavage with hemosiderin-laden macrophages. C. Bronchoalveolar lavage hemorrhagic cell block.

Fibrobronchoscopy was performed, showing a pale mucosa without lesions. Fluid from a grossly hemorrhagic bronchoalveolar lavage which highly suggested acute alveolar hemorrhage, was recovered (Fig. 3A). Cytological analysis showed 60% of alveolar macrophages with positive hemosiderin staining, 20% of lymphocytes, 20% of neutrophils, and no evidence of tumor cells (Fig. 3B and C). In microbiology studies, direct examination and cultures were negative, as well as Gen-expert.

Clinical progress was slow towards improvement, the appearance of the gluteal and trochanteric areas improved without the need of local drainage. Antibiotics were administered for 10 days, IV steroids for 3 days, and total hospital stay in the ICU was 13 days. After discharge, the patient continued with periodic outpatient controls without evidence of late sequelae neither in radiological studies nor in pulmonary function.

## 3 Discussion

Silicone embolisms, especially those involving industrial silicone used illegally for cosmetic procedures, has been implicated in various studies as a cause of acute pneumonitis with alveolar hemorrhage and ultimately of acute respiratory distress syndrome in patients undergoing esthetic procedures.^[7]^ Other local complications and adverse reactions have also been described, such as erythema, edema, abscesses, foreign body reactions that may later develop hypercalcemia and granulomatous disease.^[8–10]^ Furthermore, severe long-term autoimmune reactions secondary to silicone injection have been reported.^[11,12]^

Silicone migration produces serious systemic manifestations and mortality depends on the organ involvement, which is usually pulmonary in 82% of the cases with a mortality rate of 24% to 33%, followed by neurological affection in 18%, where the course is often fatal.^[13,14]^ It has been considered that the severity may be related to the use of adulterated or impure silicone, which increases both local and systemic adverse reactions. In the diagnostic workup, medical history and physical examination are key, as well as the events preceding symptoms onset. Imaging is used to rule out differential diagnoses such as acute pulmonary embolism associated to prolonged surgical procedures, and bronchoalveolar lavage is useful to evaluate for alveolar hemorrhage as it was seen in this case, as well as of silicone particles inside alveolar macrophages. CT-scan often shows bilateral peripheral ground glass opacities and interlobular septal thickening.^[15]^

Clinical manifestations occur in the first 24 to 48 hours after silicone injection.^[16]^ Ninety two percent present with hypoxemia, 88% with dyspnea, 78% with fever, 64% may present alveolar hemorrhage, and 33% neurological symptoms.^[2,13]^ All of these manifestations are similar to those in chemical pneumonitis, with a similar pathophysiology in which silicone phagocytosis triggers an inflammatory response, leading to lung injury.

Similar to silicone embolism is fat embolism which occurs usually after fractures to the long bones, particularly the femur, tibia, or pelvis. Schonfeld et al^[17]^ classified the severity of fat embolism syndrome through a cumulative score that includes the presence of petechiae (5 points), changes on chest x-ray (4 points), hypoxemia (3 points), tachycardia (1 point), fever (1 point), tachypnea (1 point), and confusion (1 point). A total score >5 is required for diagnosis. These characteristics can also be applied to silicone embolism.

Four patterns have been described in silicone lung injury: silicone emboli, congestion and alveolar hemorrhage, acute pneumonitis, and diffuse alveolar hemorrhage.^[5]^ Histopathological findings using infrared spectrometry reveal granulomas containing silicone vacuoles, tissue macrophages, neutrophils, eosinophils, and fibrin deposits.^[18]^ Acute silicone pneumonitis is characterized by an increase in the number of macrophages, neutrophils, and eosinophils.^[19]^

The injection of large volumes of liquid silicone directly into body tissues at high pressure can cause local tissue damage and allows the entry of the substance into the bloodstream.^[5,13]^ Subsequently, occlusion of the microvasculature occurs, causing pulmonary inflammatory response, which produces alveolar hemorrhage by increasing intravascular pressure.^[5]^ Due to the rapid evolution of events this may result in respiratory failure and death. The use of methylprednisolone boluses in the acute phase has been suggested, but there is no hard evidence to support this. Zamora et al^[6]^ described a favorable clinical course with the use of steroids and suggest that the silicone-mediated immune response plays an important role in the pathophysiology.

## 4 Conclusions

Silicone embolism incidence is rising due to its increasing use in plastic surgery, esthetic medicine, and cosmetics. Performing safe and standardized esthetic procedures avoiding the use of impure or illegal industrial compounds not suitable for human use is important to avoid complications, which range from mild respiratory symptoms to severe pneumonitis, diffuse alveolar damage, acute respiratory distress syndrome, severe alveolar hemorrhage, and central nervous system damage. When manifestations are predominantly pulmonary, patients tend to recover, but when neurological involvement predominates, mortality can rise up to 100%. A high suspicion at the time of admission, early tests to confirm the diagnosis and rule out differential diagnoses, as well as intensive care support with a multidisciplinary team are key in silicone embolism treatment.

## Author contributions

**Conceptualization:** Alejandro Bejarano, Diego F. Bautista, Luz F. Sua, Liliana Fernandez-Trujillo.

**Formal analysis:** Liliana Fernandez-Trujillo.

**Investigation:** Alejandro Bejarano, Diego F. Bautista, Liliana Fernandez-Trujillo.

**Project administration:** Liliana Fernandez-Trujillo.

**Supervision:** Diego F. Bautista, Marisol Aguirre, Liliana Fernandez-Trujillo.

**Validation:** Diego F. Bautista, Luz F. Sua, Bladimir Pérez, Juliana Lores, Marisol Aguirre, Liliana Fernandez-Trujillo.

**Visualization:** Alejandro Bejarano, Diego F. Bautista, Luz F. Sua, Bladimir Pérez, Juliana Lores, Marisol Aguirre, Liliana Fernandez-Trujillo.

**Writing – original draft:** Alejandro Bejarano, Luz F. Sua.

**Writing – review & editing:** Alejandro Bejarano, Diego F. Bautista, Luz F. Sua, Bladimir Pérez, Juliana Lores, Marisol Aguirre, Liliana Fernandez-Trujillo.
